# Lessons learnt from COVID‐19 coagulopathy

**DOI:** 10.1002/jha2.228

**Published:** 2021-05-17

**Authors:** Jecko Thachil

**Affiliations:** ^1^ Department of Haematology Manchester University Hospitals Manchester UK

**Keywords:** coagulopathy, COVID‐19, D‐dimer, fibrinogen, platelets, thrombocytopenia

## Abstract

The coronavirus disease 2019 (COVID‐19) pandemic has already left an indelible mark in human lives. Despite the havoc it created, this pandemic also saw significant advances in the management of an infectious disease wherein worldwide collaborative efforts from health care professionals have been unprecedented. One of the commonest complications recognised early in the pandemic is the development of coagulopathy. In this review, the lessons learnt from COVID‐19 coagulopathy are summarised with some perspectives on future clinical and research strategies. These include how local versus systemic coagulopathy can matter, how we can put D‐dimers to effective use, exhort more input into identifying a simple platelet activation marker, rethink the role of fibrinogen, look differently at lupus anticoagulant and heparin‐induced thrombocytopenia, bring back disseminated intravascular coagulation into our differential diagnosis slate and most importantly channel more funding into haemostasis and thrombosis research.

## 1 INTRODUCTION

The coronavirus disease 2019 (COVID‐19) caused by the severe acute respiratory syndrome coronavirus 2 (SARS‐CoV‐2) has already become the deadliest pandemic in human history, since the Spanish flu of 1918. One of the commonest causes of mortality in the millions affected by this pandemic, which is supposed to have started in Wuhan, China in December 2019, has been the very high incidence of thrombotic complications [1, 2]. To mitigate this extreme risk, several international groups suggested varying doses of anticoagulation and therefore designed various trials to incorporate these different doses [3]. When we look with great anticipation towards a ‘normal life’ when the pandemic would hopefully be brought under control with the universal use of vaccines, it is pertinent to review the lessons learnt from this pandemic from a haemostasis and thrombosis perspective.

## 2 LOCAL VERSUS SYSTEMIC COAGULATION ACTIVATION

SARS‐CoV‐2 gains access primarily through the respiratory tract. This is in keeping with the clinical findings of anosmia and lower respiratory symptoms which many patients tend to develop with this viral infection [4]. As would happen with other respiratory illnesses, the virus entry would trigger host defence mechanisms. The inflammatory response which ensues can be mild, if the viral load is low or the individual's immune response is not brisk [5]. On the other hand, the inflammatory response would be marked leading to additional complications if the viral load is high or the host response is vigorous [5]. From a coagulation perspective, a similar ‘response’ occurs with the bronchoalveolar haemostatic system [6]. This localised coagulation response exists (also in the gastrointestinal tract) to deal with invading micro‐organisms to limit their invasion into the bloodstream which would otherwise be deleterious to the host [7]. The bronchoalveolar haemostasis can thus be considered a part of the host defence similar to the immune system [8]. As much as the immune and haemostatic systems participate to control any pathogens which may have reached the airways, they tend to work together as the immunothrombosis or thromboinflammation partners [9]. A huge amount of research into these interdependent mechanisms of two fundamental physiological processes have been explored to the minutest detail [10]. What is the relevance of this liaison to COVID‐19?

**FIGURE 1:**
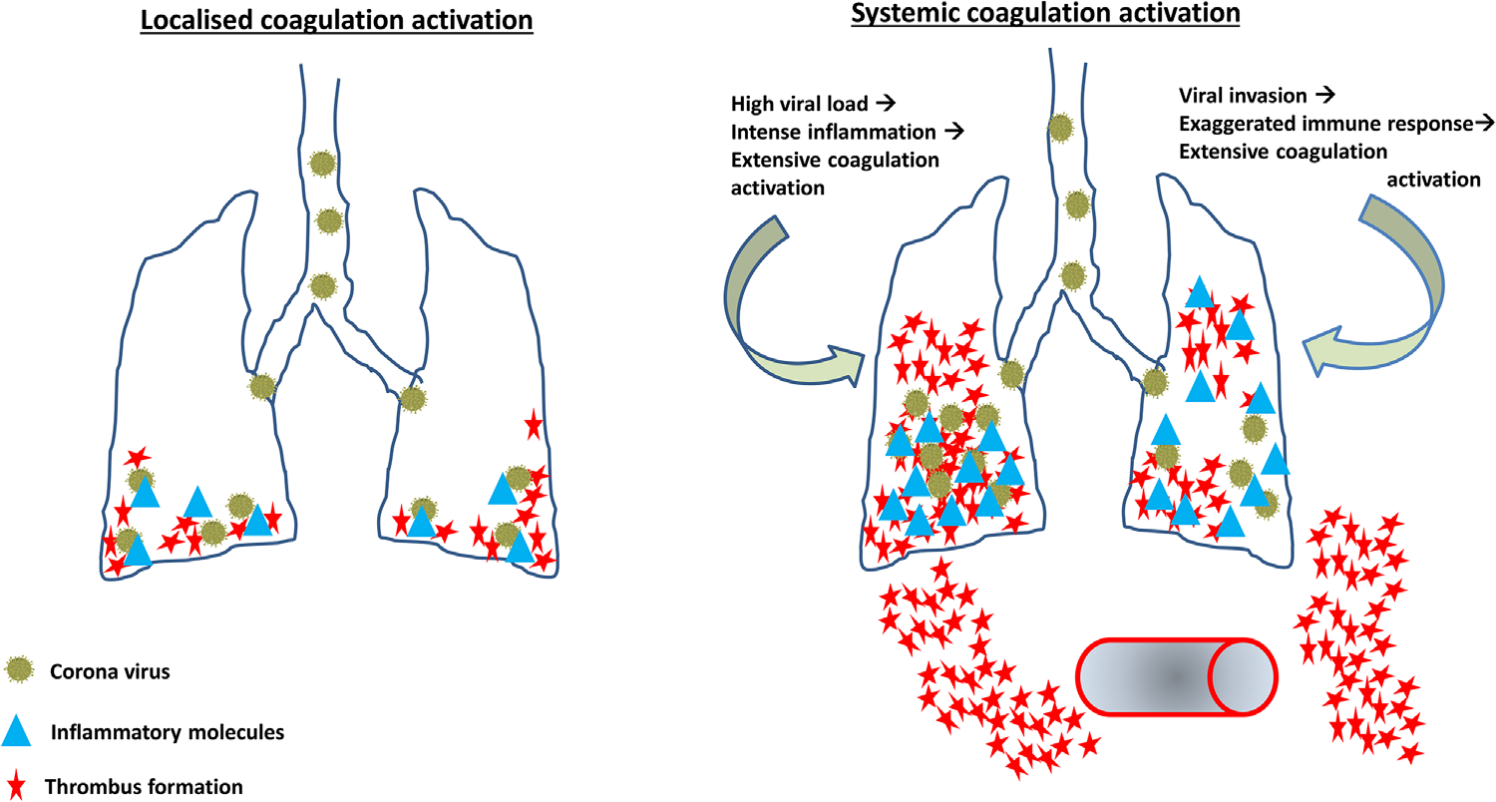
Differentiating localised versus systemic coagulation activation in COVID‐19. In localised coagulation activation (left of the figure), viral invasion activates the host immune and coagulation systems. There is also interplay between the host systems termed immunothrombosis. Two situations with the viral infection can cause systemic coagulation activation – first, if there is a high viral load, it causes heightened immune response and thus extensive coagulation activation which spreads into the circulation to cause thrombi in the different circulatory beds. Similar dissemination of the coagulation can happen with moderate viral load but if the host immune response is aggressive

- Localised thrombi formation in the lungs may represent a positive host response and may not require treatment in the absence of respiratory compromise. This has already been demonstrated in studies where the pulmonary thrombus load in COVID‐19 patients (compared with non‐COVID‐19) is lower with the distribution of the clots being more in the peripheral pulmonary arteries [11]. This is akin to the finding of incidental pulmonary thrombi in patients with cancers [12].
- If the hosts’ immune response is effective in clearing the virus, the vigorous fibrinolytic system in the alveolar environment can break down the localised clots and re‐enable normal gas exchange. This is likely to have happened in many individuals who did not require hospitalisation with the infection (see Figure 1).
- Continuing, unablated inflammation on the other hand can lead to pulmonary thrombi formation which may then disseminate into the systemic circulation and cause thrombi in various circulatory beds [13]. These thrombi can clinically manifest as cerebral or peripheral limb ischaemia, venous thrombi in the lower limbs and clots in the splanchnic vascular beds.
- Early control of inflammation and thus of the thromboinflammatory process may be beneficial by preventing significant thrombosis. Reduced mortality was observed by the Dutch investigators in the second wave compared with the first wave despite an unchanged high incidence of thrombotic complications suggesting better and early control of the disease process could mean decreased mortality from significant thrombosis [14].
- Future research should thus look at how localised thromboinflammation in the lungs and gut may be beneficial and at what stage this beneficial effect can change (progress?) to become harmful. An interesting area of research in this respect is the role of inhaled heparin for lung thromboinflammation and oral anti‐inflammatory, anticoagulant (DOAC?) in gut thromboinflammation.

## 3 D‐DIMERS AS A NON‐THROMBOTIC LABORATORY MARKER

Until this pandemic, D‐dimers had been commonly used as a laboratory test to exclude venous thromboembolism in those patients with a low clinical probability for such a diagnosis [15]. D‐dimers were never intended to become a diagnostic test for VTE but sadly, this has become the common practice since the pandemic. A very high incidence of thrombosis in these patients with the only consistent abnormal coagulation marker being the elevated D‐dimers led to many health care professionals to use the test as THE test to determine thrombotic risk [16]. In the authors clinical practice, D‐dimers were very rarely requested in the paediatric patients but since the pandemic, with the recognition of the rare PIMS‐TS (paediatric inflammatory multisystem syndrome, temporally associated with SARS‐CoV‐2), this test is now required in the order panel of any child presenting with fever and respiratory symptoms to a hospital [17].

D‐dimers are indeed the breakdown products of cross‐linked fibrin during the process of fibrinolysis [15]. However, D‐dimers are increased in many diseases; only one of them being thrombus formation. Most of the review publications about D‐dimers give a list of several conditions where this laboratory test Increased in the absence of VTE and advise caution in the interpretation of results if these conditions were present [18]. So how can we turn the misuse of this laboratory test into one that can be aiding us in the future?

- D‐dimers have consistently been shown to be a prognostic marker in COVID‐19 [19, 20]. This predictive capacity is independent of the heightened thrombotic risk suggesting it may signify the inflammatory process which can generate D‐dimers from extravascular fibrinolysis [12, 21].
- D‐dimers also correlate with acute lung injury in COVID‐19; a complication which can increase the need for mechanical ventilation and mortality [22]. It may be worthwhile studying serial D‐dimers in predicting acute lung injury in other causes of acute lung injury like sepsis or trauma. A worsening D‐dimer may mean more aggressive measures to be considered to prevent acute lung injury while stable or improving D‐dimers mean time to consider tapering the treatment modalities.
- One of the unusual manifestations of COVID‐19 is the paediatric inflammatory multisystem syndrome temporally associated with SARS‐CoV‐2 (PIMS‐TS). D‐dimers are markedly raised in these children in tandem with the other inflammatory markers [23]. This finding could suggest D‐dimer can be used as an inflammatory marker in vasculitides and rheumatological disorders where there may be leakage of plasma proteins into extravascular space. D‐dimers may be particularly useful since the currently available diagnostic tests cannot point to this pathophysiological process of vascular leakage.

## 4 NEED FOR A SIMPLE LABORATORY MARKER FOR PLATELET ACTIVATION

Platelets have a wide repertoire of functions which includes antimicrobial properties [24]. This anti‐pathogen role is often used to explain the thrombocytopenia which develops in patients with infections whereby the platelets play a suicidal role in their efforts to kill the pathogen on the one hand [25]. On the other hand, the pathogen may try to escape detection from the host's immune surveillance by entering and hiding inside the platelets, which then get culled by the spleen because they do not look normal anymore [26]. Despite its useful viral attack role, it is quite surprising that COVID‐19 does not cause marked thrombocytopenia [27]. The lowest platelet counts recorded are in the 40–50 × 10^9^/L with most patients with even severe COVID‐19 having only a mild thrombocytopenia [28]. Although thrombocytopenia has been rare, there has been a large amount of basic science research into the role of platelets in COVID‐19 [29, 30].

Various mechanisms have been suggested for platelets participation in COVID‐19 [31, 32, 33, 34]. These elegant studies point to the very clear message – platelets are hyper‐reactive in COVID‐19. However, the dilemma is how much of these super‐active platelets are performing functions which are aimed at protecting the patients from COVID‐19 and what proportion of the increased platelet reactivity is causing harm by forming platelet aggregates and leading on to microthrombi and macrothrombi. The crucial question is how we can identify the time window when the beneficial platelet may turn to become harmful for the simple reason that this is when we can intervene with antiplatelet therapies and reap maximum benefits [35]. Timing is important – an early intervention with such drugs may dampen the beneficial role of the platelets while the late approach would simply be too late [36, 37]. Another conundrum in this regard is the need to understand the predominant platelet pathways responsible for the beneficial and harmful effects? Knowledge of the specific signalling pathways would indeed be necessary to design newer antiplatelet drugs or tailor currently available therapies to the best effects. For example, it has been suggested that aspirin may potentiate the inflammatory response which is characteristic of COVID‐19 while P2Y12 inhibitors may dampen it [38, 39]. The specific P2Y12 inhibitor, ticagrelor can stimulate neutrophil chemotaxis and phagocytosis and beneficially influence endothelial nitric oxide function, while clopidogrel can have an impact on cytokines but does not counteract endothelial nitric oxide depletion [39]. What do all these brilliant laboratory works mean for clinicians who want to translate such incredible science into clinical practice?

**FIGURE 2:**
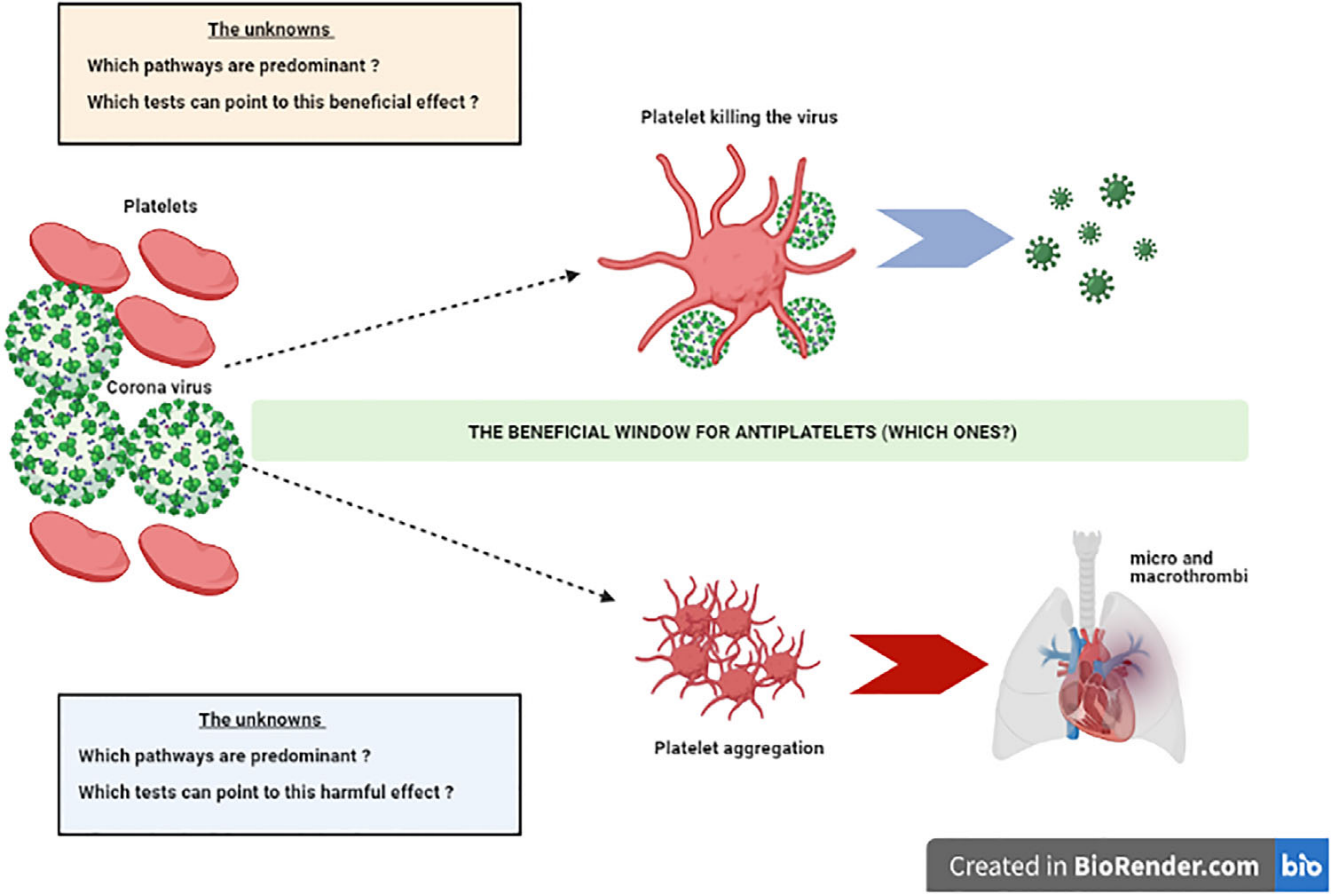
Platelet activation in COVID19. The severe acute respiratory syndrome coronavirus 2 has been shown to activate platelets in different studies. Platelet activation may be beneficial to an extent to limit the viral invasion but in other circumstances may be harmful by causing thrombi through platelet aggregation. What is crucial in these situations is to understand the key mechanisms involved so that appropriate platelet‐focused therapies can be instituted. Timing of such treatments is paramount to reap maximum benefits

- There is an urgent need to identify simpler markers for platelet activation which can be used in the mainstream clinical practice. Currently, the only ‘available’ laboratory test for platelet function is the platelet count and possibly mean platelet volume which is yet to be standardised and widely accepted [40].
- Non‐haematologists need to be aware that platelets are more than just a haemostatic cell. They have several functions which play a relevant role in infection and inflammation.
- A key aspect of platelet research is to translate the experimental findings to effective therapeutic measures. It is very likely that multiple signalling pathways are involved in disease processes and trying to target them with ‘broad‐spectrum’ antiplatelets like aspirin or signal‐specific antiplatelets like P_2_Y_12_ inhibitors may not always be successful (Figure 2).
- Even when we may identify interesting therapeutic targets to manipulate platelet function, the crucial part is to identify the timing of their administration since if we were to dispense targeted therapy when the platelet activation is playing a beneficial role, then it could result in negative outcomes and vice versa (Figure 2).

## 5 FIBRINOGEN – FRIEND OR FOE OR BOTH?

Being the first discovered clotting factor and rightly named coagulation factor I, fibrinogen plays a very important role in haemostasis and thrombosis [41]. But, unfortunately, haematologists only ‘think’ about fibrinogen when a patient is bleeding heavily. It is well known that fibrinogen increases in inflammatory states being an acute phase reactant [42]. In addition, there is also the physiological increase in clinical states like pregnancy. Although this acute phase response is well recognised, the role this response plays in pregnancy and inflammatory states still remains a mystery.

Participation of fibrinogen in anti‐infective process is evident in the fact that although it is primarily synthesised in the liver under normal conditions, the production also occurs in the lung and intestinal epithelium (areas which are commonly exposed to infections) under inflammatory states [43, 44]. In bacterial infections, the microbes have been shown to express or release fibrinogen binding proteins (e.g., streptococcal M1 protein) in order to envelope themselves from host immune recognition [45]. A very recent, elegant study demonstrated a protective effect for fibrinogen in inflammatory states [46]. Fibrinogen and fibrin protected cells from the cytotoxic effects of histones, which are released in tissue damage states [46]. Fibrinogen but not fibrin delayed the process of NETosis (neutrophil extracellular trap formation) triggered by ionomycin in the experimental setting [46]. It is useful to bear in the mind that the demonstrable increase in fibrinogen in the clinical scenarios where it behaves as an acute phase reactant also have simultaneous increase of tissue damage proteins which can cause host endothelial damage [47]. The increased levels of fibrinogen can bind to the large amounts of histones and (possibly other tissue damage proteins) to complex it away and thus protect the host from adverse outcome [47]. On the same note, once fibrinogen starts getting ‘used up’ by getting consumed in the process of quenching cell damage molecules and simultaneously participate in thrombus formation (most inflammatory conditions cause thrombosis). If there was a shortage of fibrinogen, having been consumed into clots, the uncomplexed damage‐associated proteins could cause endothelial damage which would manifest as disseminated intravascular coagulation. In COVID‐19, marked hyper‐fibrinogenemia was noted in most studies but none of these publications reported a correlation for the markedly elevated fibrinogen levels to worse outcomes [48]. What was indeed observed was the worse outcomes when the fibrinogen levels started decreasing [49]. How can we put together the messages learnt regarding fibrinogen thus far?

- High levels of fibrinogen, as an acute phase reactant, may be protective by forming complexes with tissue damage proteins and may not necessarily be a risk factor for thrombosis. Serially monitoring fibrinogen in these circumstances can be helpful since a downward trend portend poor prognosis.
- More research is needed on the non‐haemostatic functions of fibrinogen.
- Another interesting aspect: can fibrinogen behave as an antimicrobial protein? One of the reasons this aspect has not been so far explored is probably due to the use of serum instead of plasma in research studies examining antimicrobial substances in the body.
- Can we create a non‐haemostatic fibrinogen which can bind damage‐associated molecules and not participate in thrombosis? This would be similar to the research focusing on the non‐anticoagulant properties of heparin which are being actively searched for commercial purposes including in the anti‐cancer setting. This approach would benefit from a therapeutic target which cause no harm like thrombosis (akin to anti‐factor XI and XII molecules being trialled as anti‐thrombotic but with no risk of bleeding).

## 6 THE LUPUS ANTICOAGULANT CONUNDRUM

Early on in the pandemic, multiple reports were published on the positive lupus anticoagulant in patients with COVID‐19 [50, 51]. This caused a bit of an uproar since several experts were sceptical about the appropriateness of looking for lupus anticoagulant outside the setting of antiphospholipid syndrome (APS). In addition, transient lupus anticoagulant positivity has always been considered an epiphenomenon with no real link to any untoward consequences including arterial or venous thrombosis. But can lupus anticoagulant positivity mean something?

Lupus anticoagulant is mostly caused by anti‐phospholipid antibodies which include anticardiolipin antibodies and beta‐2‐glycoprotein antibodies [52]. Common finding of lupus anticoagulant in COVID‐19 led some investigators to examine the role of these antibodies in the viral infection. Antiphospholipid antibodies from patients with COVID‐19 was more reactive towards beta‐2‐glycoprotein‐coated plates in comparison with cardiolipin‐coated ones and were of low to medium titres when they are of medium/high titres in APS patients [53]. This variation was because of specificity of the antibodies to C‐terminal domains 4–5 instead of N‐terminal domain 1; this particular specificity for the N‐terminal domain of beta‐2‐glycoprotein being strongly linked to the risk of thrombosis and pregnancy complications in APS [53]. Although low titre antibodies are not a thrombotic risk, an interesting proposition by the authors of this publication was that β2GPI may accumulate in high density on activated endothelium and trigger thrombus formation [53, 54].

- Does this mean lupus anticoagulant positivity with localised inflammation (obstetrics, vascular inflammation in cerebral or coronary vessel) carry a thrombotic risk?
- Should we be giving importance to even transient lupus anticoagulant positivity in acute inflammatory settings and monitor their persistence or absence to risk stratify patients’ thrombotic risk?

## 7 A NEW ‘HIT’

Another immune problem which was observed in the COVID‐19 coagulopathy setting is the well‐known therapeutic complication of heparin‐induced thrombocytopenia (HIT). Although this adverse effect with the use of heparin can occur in COVID‐19 patients as much as in the pre‐COVID‐19 era with the widespread use of heparin, HIT incidence has been suggested to be much higher than in the critically ill non‐COVID‐19 patients [55, 56]. Nazy et al recently described findings in 10 critically ill COVID‐19 patients who developed thrombocytopenia while receiving heparin and thus a clinical suspicion for HIT was raised [57]. These patients were noted to have immune complexes capable of mediating platelet activation and thus thrombocytopenia but in contrast to HIT, these complexes did not include anti‐PF4/heparin antibodies. Such an immune mediated activation of platelets was also shown by another group who demonstrated immunoglobulin G fractions from COVID‐19 patients are able to cause platelet apoptosis and increased phosphatidyl serine externalisation; thus, procoagulant platelets [58]. Such a phenotype was also induced by immune complexes and not just antibodies similar to that noted in lupus vasculitis [58, 59].

A lot of interest has been raised in the last few weeks about a very rare atypical HIT with the description of vaccine‐induced immune thrombotic thrombocytopenia (VITT) [60, 61]. In this syndrome, antibodies, similar to HIT antibodies, but without prior heparin exposure, develop with certain types of COVID‐19 vaccination. These antibodies cause severe thrombocytopenia and thrombosis (including the unusual cerebral venous thrombosis) [60, 61]. What is intriguing is why such intense immune activation occurs against platelets when it is relatively rare with the other blood cells?

These observations on HIT and VITT make us think:

- does atypical HIT occur more commonly in critically ill patients and we have not been searching for it?
- does thrombocytopenia associated with immunological disorders (e.g., systemic lupus erythematosus) have a similar underlying pathophysiological mechanism to that of atypical HIT?
- do catastrophic APS, VITT and maybe some resistant cases of thrombotic microangiopathies (e.g., related to stem cell transplant or drugs) have a VITT‐like pathophysiology?

## 8 DOES DIC EXIST IN COVID‐19 OR ANY OTHER ILLNESSES (ANYMORE)?

One of the earliest publications including COVID‐19 patients discussed the development of disseminated intravascular coagulation (DIC) in severe cases and was accompanied by an editorial by a respected author [49, 62]. However, subsequent literature has categorically denied any evidence for DIC in SARS Co‐V 2 infection [63]. In the authors opinion, considering DIC to be a non‐entity in this pandemic is because of a common misunderstanding about the laboratory parameters of DIC [64]. Most clinicians discount DIC as the diagnosis when the prothrombin time is normal, and fibrinogen is not markedly reduced. Besides, DIC diagnosis is not entertained in the absence of widespread bleeding or acral ischaemia, which is very rare in many infections in the 21st century because of early diagnosis and intervention [65]. The subcommittee on DIC of the ISTH has defined DIC as ‘An acquired syndrome characterised by the intravascular activation of coagulation with loss of localisation arising from different causes. It can originate from and cause damage to the microvasculature, which if sufficiently severe, can produce organ dysfunction’. [66]. There are several aspects of this definition which is worth stressing. First, it is intravascular coagulation; a process which is almost always pathological. Second, there is loss of localisation due to the dysregulated thrombin generation which overwhelms the endogenous anticoagulants and other antithrombotic checks. If we take the example of COVID‐19, this dissemination does occur in some patients where the clot formation is not limited to the lungs, and different organs are affected. The third crucial part of the DIC definition is the role of the vascular endothelium, the most ubiquitous organ in human body. If marked endothelial dysfunction results in organ damage from microthrombi, it could be considered DIC according to the ISTH definition. In the authors’ opinion, the third concept has been re‐termed ‘coagulopathy’ in the 21st century, which seems to be DIC with an attractive nickname. For example, coagulation disturbances due to sepsis have been re‐termed ‘sepsis‐induced coagulopathy’ while that associated with trauma is now widely known as ‘acute trauma coagulopathy’ or ‘trauma‐induced coagulopathy’.

**FIGURE 3:**
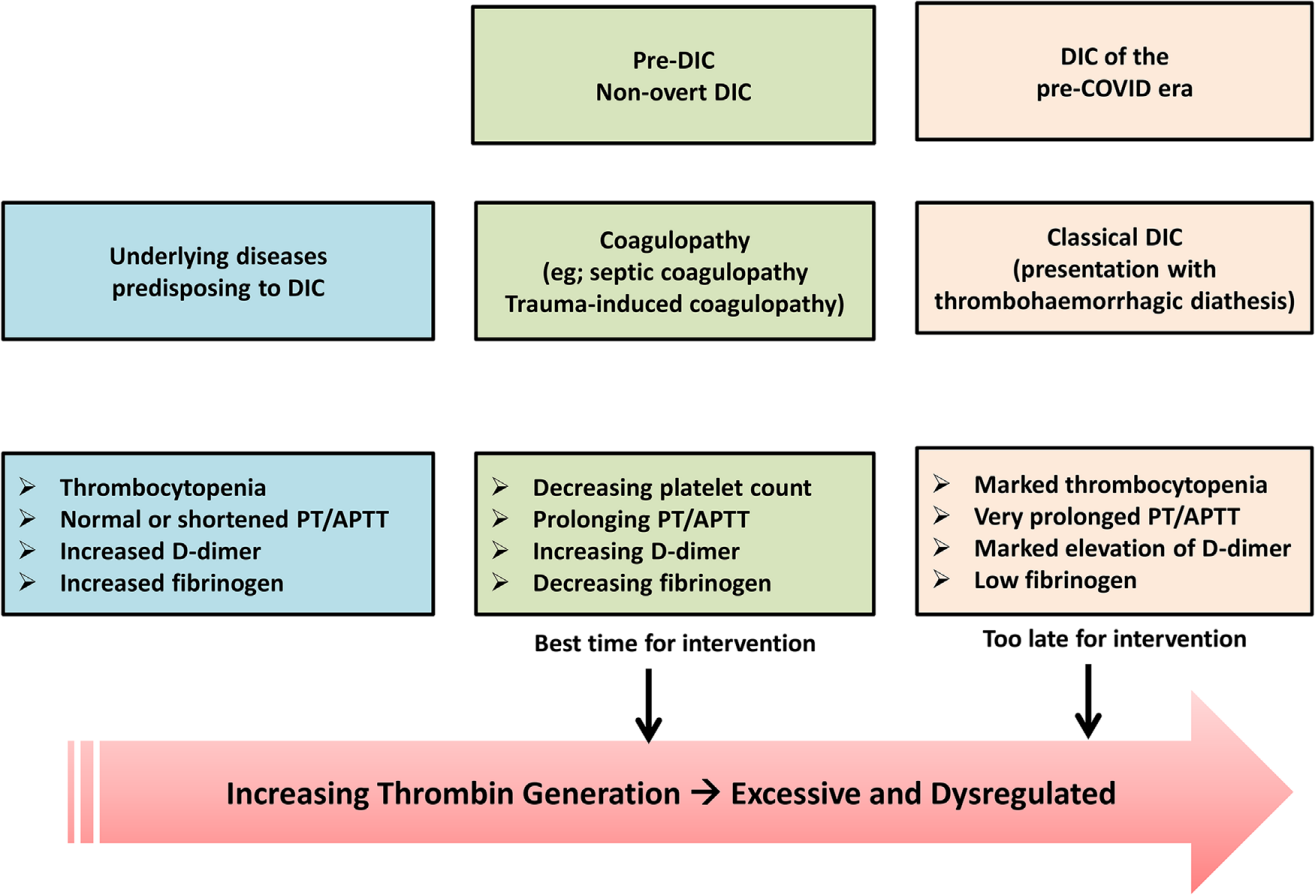
Disseminated intravascular coagulation in 2021. In the presence of an underlying condition which can cause DIC, a combination of worsening laboratory parameters can be considered to be DIC but currently is considered as coagulopathy. This stage (middle section in the figure) is the best time for intervention and may be considered as non‐overt DIC. However, DIC is often entertained when the lab results are extremely deranged when the interventions may be futile

So, can we make a diagnosis of DIC earlier than in pre‐death situations? A simple method in this respect is to monitor the laboratory markers for DIC since one set of blood samples only provides a snapshot of the DIC process. Monitoring these tests is necessary to understand DIC process which is a continuum. In other words, DIC diagnosis should be considered when the platelet count starts dropping (not when it is too low), PT starts prolonging (not when it is markedly prolonged) and when the fibrinogen starts decreasing (not when it may be less than 1.5–2 g/L) and the D‐dimers start getting worse [67, 68] (Figure 3). Once these blood markers stop worsening, de‐escalating anti‐DIC interventions may be considered. Relevance of monitoring the laboratory parameters in DIC was stressed in the British Society of Haematology guidelines over 10 years ago [68]. Despite this, many publications alluding to DIC only provide a single set of results as representative of DIC. This single end‐point focus has possibly led to the downfall of what may have been effective therapies like thrombomodulin and antithrombin.

## 9 URGENT NEED FOR FUNDING NON‐MALIGNANT HAEMATOLOGY RESEARCH

Since the 20th century, progress in any field including medical research would highly depend on adequate funding. In the specialist field of haematology, focus has been on research into better diagnosis and treatment of malignant diseases which certainly deserve the attention from the public, media and researchers alike. However, COVID‐19 has brought an awakening in this respect. As a global nation, we understood how a very minute virus could bring us to our knees unless we are better prepared and vigilant. Thrombotic complications in this pandemic have marshalled research troops all over the world to work together on decreasing mortality from this infection. Similar to COVID‐19, many bacterial, viral and parasitic infections continue to cause high mortality by causing bleeding or thrombosis. It is high time that funding agencies; both pharmaceutical and governmental bodies provide grants towards basic science and clinical research in these areas. Let us hope the downside of COVID‐19 has some upside in this aspect.

## 10 CONCLUSION

In summary, COVID‐19 has taught us a lot about the various aspects in the field of haemostasis and thrombosis. We stood on the shoulders of the fascinating research work which was carrying on in the pre‐COVID‐19 era which helped tremendously in the management of haemostatic complications during the pandemic. We can also feel good about the fact that COVID‐19 brought us even closer in the field of collaborative work to advance science and therapeutics and limit the mortality from this viral pandemic. It is certain this concerted effort will continue and work towards the goal where we can confidently deal with any thrombotic issues with confidence in the future.

## CONFLICTS OF INTEREST

The authors declare no conflict of interest.
